# High-density genetic map construction and identification of loci controlling flower-type traits in Chrysanthemum (*Chrysanthemum* × *morifolium* Ramat.)

**DOI:** 10.1038/s41438-020-0333-1

**Published:** 2020-07-01

**Authors:** Xuebin Song, Yuhui Xu, Kang Gao, Guangxun Fan, Fan Zhang, Chengyan Deng, Silan Dai, He Huang, Huaigen Xin, Yingying Li

**Affiliations:** 1 Beijing Advanced Innovation Center for Tree Breeding by Molecular Design, Beijing Key Laboratory of Ornamental Plants Germplasm Innovation & Molecular Breeding, National Engineering Research Center for Floriculture, Beijing Laboratory of Urban and Rural Ecological Environment, Key Laboratory of Genetics and Breeding in Forest Trees and Ornamental Plants of the Ministry of Education, Beijing Forestry University, School of Landscape Architecture, Beijing Forestry University, 35 East Qinghua Road, Beijing, 100083 China; 2 College of Landscape Architecture and Forestry, Qingdao Agricultural University, Qingdao, 266109 Shandong China; 3 Biomarker Technologies Co., LTD, Beijing, 101300 China; 4 LC Science Co., LTD., Hangzhou, 310018 China

**Keywords:** Plant hybridization, Genetic variation

## Abstract

Flower type is an important and extremely complicated trait of chrysanthemum. The corolla tube merged degree (CTMD) and the relative number of ray florets (RNRF) are the two key factors affecting chrysanthemum flower type. However, few reports have clarified the inheritance of these two complex traits, which limits directed breeding for flower-type improvement. In this study, 305 F_1_ hybrids were obtained from two parents with obvious differences in CTMD and RNRF performance. Using specific-locus amplified fragment sequencing (SLAF-seq) technology, we constructed a high-density genetic linkage map with an average map distance of 0.76 cM. Three major QTLs controlling CTMD and four major QTLs underlying RNRF were repeatedly detected in the 2 years. Moreover, the synteny between the genetic map and other Compositae species was investigated, and weak collinearity was observed. In QTL regions with a high degree of genomic collinearity, eight annotated genes were probed in the *Helianthus annuus* L. and *Lactuca sativa* L. var. ramosa Hort. genomes. Furthermore, 20 and 11 unigenes were identified via BLAST searches between the SNP markers of the QTL regions and the *C. vestitum* and *C. lavandulifolium* transcriptomes, respectively. These results lay a foundation for molecular marker-assisted breeding and candidate gene exploration in chrysanthemum without a reference assembly.

## Introduction

Flower type is an important quality trait of all ornamental plants^1,2^. Flower-type variation is extremely abundant in chrysanthemum due to the presence of ray and disc florets with complex and varied shapes in the chrysanthemum capitulum. The composition of different chrysanthemum types is also intricate, in which the morphology of florets (referred to as petal type in previous studies) and the relative number of ray florets (relative to disc florets, referred to as flower doubleness in previous studies) are two key traits affecting the chrysanthemum type^3,4^. However, directional breeding aimed at shaping flower types is an enormous challenge because of the limited understanding of the molecular mechanisms of flower-type development. In a previous study, we defined flower doubleness as the relative number of ray florets (RNRF) and the ray floret shape as the corolla tube merged degree (CTMD). Moreover, we found that these two complex traits are quantitative traits, supported by major gene and polygene models^5^. In chrysanthemum breeding practice, cultivars with greater flower doubleness and a higher degree of tubular structure in the ray florets always present higher ornamental value due to their long-lasting flowering and long postharvest storage time^6^. Therefore, it is of practical significance to analyze the genetic mechanisms of these two complex traits to breed new varieties with higher ornamental value.

QTL analysis based on genetic linkage maps is a valuable and effective method for mining tightly linked molecular markers and determining the positions of genes controlling complex quantitative traits^7,8^. Peltier constructed the first genetic map of petunia in 1994. Since then, genetic maps of at least 27 ornamental plants have been constructed (Supplementary Fig. 1). Most of these maps were based on traditional markers such as AFLPs and SSRs, and their mapping resolution is limited because of the relatively small number of available molecular markers^9^. Recently, SNP markers identified based on next-generation sequencing (NGS) have been considered ideal molecular marker types for high-density genetic map construction. Restriction enzyme-assisted sequencing methods, including restriction-site-associated DNA sequencing (RAD-seq^10^), genotyping by sequencing (GBS^11^) and specific-locus amplified fragment sequencing (SLAF-seq^12^), are a less costly strategy using NGS technology that enables the development of SNP markers at the genome-wide level, even for polyploid plants with high heterozygosity and without reference genomes. SLAF-seq has provided the possibility of ultrahigh-density genetic map construction and fine mapping in ornamental plants with complex genetic backgrounds^13–16^. In addition, due to this complexity, researchers often use a double-pseudo-testcross mapping strategy to construct genetic maps of ornamental plants. Based on SLAF-seq technology, 204,361 SNP markers were developed in F_1_*Ginkgo biloba* plants, and a high-density genetic map with 12,263 markers was constructed (the total length of the map was 1671.77 cM, and the average map distance was 0.89 cM). Compared with the framework linkage map constructed based on SSR, SRAP and AFLP markers, the above markers added more than 10,000 markers, and the average map distance was reduced by 9.93 cM (compared to 10.82 cM with the shortest average distance between markers)^17^. This strategy is particularly advantageous for the construction of maps in highly heterozygous ornamental plants without reference genomes. Based on this technology, ultrahigh-density genetic maps with an average map distance of less than 1 cM have been successfully constructed for *Paeonia suffruticosa*^18^, *Osmanthus fragrans*^19^ and *Prunus mume*^14^. This technique can also be applied to many large and complex polyploid genomes. For example, researchers^20,21^ have developed tens of thousands or even hundreds of thousands of high-quality SNP markers in hexaploid sweet potatoes based on this technology. The development efficiency of this approach is much higher than that of other molecular marker technologies (e.g., SRAP, SSR and AFLP). Moreover, SNP markers obtained from the whole genome can promote the subsequent analysis of population evolution and fine mapping^22^.

Chrysanthemum is a self-incompatible ornamental plant with a highly heterozygous genome, and most cultivars are hexaploid or aneuploid^23,24^. Therefore, the construction of high-density genetic maps for chrysanthemum has always been an enormous challenge. Only two genetic maps have been published to date. Based on 142 individuals from an F_1_ population, a framework genetic map for chrysanthemum containing 597 of 1067 markers (153 RAPDs, 61 ISSRs, 353 AFLPs and 500 SRAPs) was constructed^25^. The total lengths of the genetic maps for the female and male parents were 1912.8 and 1887.9 cM, respectively, and the average distances between adjacent markers were 6.9 and 6.6 cM, respectively. Based in the genetic map, some QTLs related to flower diameter, the number of ray florets, the number of disc florets, ray floret length, disc floret length, disc floret width and the center diameter of disc florets were identified^26^. Then, a high-density genetic map for chrysanthemum was constructed including 30,312 SNPs^27^. The genetic distance of the obtained linkage groups ranged from 64.5 to 95 cM, and the total length of the map was 752.1 cM. QTLs associated with flower color, flowering time, disk floret degreening and the number of ray florets were also mapped^27^. However, high-resolution QTLs responsible for the two key traits (CTMD and RNRF) related to chrysanthemum flower type have not yet been discovered.

According to a double-pseudotestcross strategy, we used SLAF-seq technology to develop SNP markers and constructed a high-density genetic linkage map for chrysanthemum. Then, QTL analysis was performed to illuminate the genetic mechanisms underlying CTMD and RNRF over the course of 2 years. To further screen possible candidate genes underlying major QTLs, we mapped the SNP markers to the reference genome sequences of sunflower^28^ and lettuce^29^ to search the corresponding genes sharing genomic syntenic segments. In addition, the SNP makers within QTL regions were mapped to two wild chrysanthemum unigene libraries developed by RNA-seq. This study provides a foundation of selective molecular markers for marker-assisted breeding and the gene mapping and map cloning of QTLs for CTMD and RNRF in chrysanthemum.

## Results

### Evaluation of SLAF sequencing quality and genotyping

High-throughput DNA sequencing generated a total of 406.28 GB of raw data with a Q30 of 92.42% and a GC content of 40.47% (Table 1). The digestion efficiency was 95.81%, and a total of 2,033,900,854 paired-end reads of 150 bp in length were obtained. Among these reads, 905,428 SNPs were detected, for which the average sequencing depth was 59.69 X in “225”, 57.48 X in “Candy”, and 23.52 X in each of the F_1_ offspring. Among these SNPs, 234,648 were successfully genotyped, and 214,882 could be used for genetic map construction. In addition, 26,085 polymorphic SNP markers were identified, accounting for 12.14% of the markers used in genetic mapping. Among these markers, 9401 high-quality SNPs could be used to construct the chrysanthemum genetic map, including the lm×11 (74), nn×np (4548) and hk×hk (4419) types.

**Table 1:** Statistical sequencing data for the two parents and F_1_ offspring

Sample ID	Total reads	Total bases	SLAF numbers	Average depth	Polyslaf	Q30 percentage (%)	GC percentage (%)
Male ‘225’	18,621,440	3,712,776,506	396,130	57.32×	131,193	93.32	41.07
Female ‘Candy’	18,004,773	3,590,276,560	395,411	59.14×	130,419	93.10	41.02
Offspring	6,548,441.45	1,308,127,346.51	269,455	18.05×	82,244	92.41	40.47
Control	1,019,501	203,670,506	/	/	/	92.33	45.00
Total	2,033,900,854	406,281,893,752	612,325	18.32×	149,209	92.42	40.47

### High-density genetic map construction

**Fig. 1:**
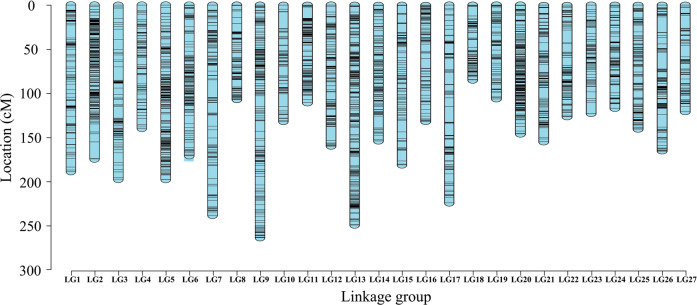
High-density genetic map of chrysanthemum based on 6452 SNPs. The abscissa is the linkage group and the ordinate is the location

By calculating the mLOD values between the two high-quality SNPs, a total of 3218 markers were developed for the genetic map of the female parent ‘Candy’, with a total genetic map distance of 3758.92 cM and LG lengths ranging from 0 to 290.86 cM (Supplementary Table 1-1). A total of 3410 markers were developed for the genetic map of the male parent ‘225’, with a total genetic map distance of 3693.23 cM and LG lengths ranging from 0 to 270.53 cM (Supplementary Tables 1 and 2). On the basis of the collinear sites in every LG, the final map containing 6452 markers was constructed by the integration of the parents’ genetic maps (Table 2 and Fig. 1). The final map was 4301.5 cM in length with an average distance of 0.76 cM. The length of the LGs ranged from 84.66 cM (LG18) to 263.37 cM (LG9). On average, the LGs were 159.315 cM in length and contained 270 markers spanning 120.84 cM. The number of markers in the map ranged from 124 (LG3, average distance of 1.59 cM) to 528 (LG2, average distance of 0.33 cM). The longest linkage group was LG9, which contained 299 markers and exhibited a genetic map distance of 263.37 cM and an average intermarker distance of 0.88 cM. The shortest linkage group was LG18, which contained 147 markers and exhibited a genetic map distance of 84.66 cM and an average intermarker distance of 0.58 cM. The largest genetic gap was found in LG2, which was 18.92 cM in length, and the smallest gap was found in LG20, which was 5.23 cM in length.

**Table 2:** Description of the basic characteristics of the 27 linkage groups

Linkage group	Total markers	Total distance (cM)	Average distance (cM)	Gaps <5 cM (%)	Max gap (cM)
LG1	136	188.58	1.39	91.85	14.72
LG2	528	174.22	0.33	99.43	18.92
LG3	124	197.19	1.59	89.43	15.21
LG4	138	139.51	1.01	94.89	10.14
LG5	442	197.53	0.45	99.32	7.38
LG6	291	170.57	0.59	98.28	9.35
LG7	251	238.09	0.95	95.2	19.87
LG8	183	106.84	0.58	97.25	8.81
LG9	299	263.37	0.88	97.65	12.37
LG10	138	131.94	0.96	95.62	18.9
LG11	211	110.45	0.52	98.57	12.56
LG12	256	159.75	0.62	97.65	15.66
LG13	516	249.09	0.48	98.45	10.12
LG14	255	153.84	0.6	99.21	13.37
LG15	206	180.95	0.88	95.61	12.66
LG16	185	131.81	0.71	97.28	10.95
LG17	249	223.9	0.9	93.15	17.48
LG18	147	84.66	0.58	97.95	10.99
LG19	170	106.31	0.63	97.04	5.58
LG20	475	145.79	0.31	99.79	5.23
LG21	219	154.69	0.71	97.25	11.95
LG22	166	126.38	0.76	96.97	14.55
LG23	128	122.63	0.96	96.06	12.61
LG24	151	116.89	0.77	96.67	8.95
LG25	240	140.43	0.59	97.49	7.79
LG26	189	165.43	0.88	95.74	18.9
LG27	159	120.66	0.76	96.84	14.3
Total	6452	4301.50	0.76	96.69	19.87

According to a chi-square test of the 6452 markers, there were 525 markers showing segregation distortion (*P* < 0.05) even though the extremely significant SNPs (*P* < 0.01) were excluded anteriorly, and the total segregation distortion ratio was 8.14%. The greatest number of segregation distortion markers occurred in LG11, with a segregation distortion ratio of 35.55%. There were no markers of segregation distortion in LG13 (Table 3).

**Table 3:** Distribution of distorted segregation markers in parental and integrated genetic maps

Linkage group	Total markers	Number of markers of segregation distortion	Segregation distortion ratio (%)
LG01	136	23	16.91
LG02	528	30	5.68
LG03	124	2	1.61
LG04	138	12	8.70
LG05	442	24	5.43
LG06	291	25	8.59
LG07	251	12	4.78
LG08	183	9	4.92
LG09	299	29	9.70
LG10	138	30	21.74
LG11	211	75	35.55
LG12	256	15	5.86
LG13	515	0	0.00
LG14	255	3	1.18
LG15	206	34	16.50
LG16	185	16	8.65
LG17	249	24	9.64
LG18	147	4	2.72
LG19	170	2	1.18
LG20	475	42	8.84
LG21	219	27	12.33
LG22	166	7	4.22
LG23	128	5	3.91
LG24	151	6	3.97
LG25	240	7	2.92
LG26	189	29	15.34
LG27	159	33	20.75
Total	6451	525	8.14

### SNP verification

To verify the accuracy of the SNPs in SLAF markers in the genetic map, we genotyped 80 individuals using KASP technology. Among the 15 pairs of SNPs selected, 10 were successfully genotyped in most individuals, with a success rate of 95% (successfully genotyped points/total number of points), indicating a high accuracy of the SLAF markers in our genetic map.

### QTL analysis of RNRF and CTMD

A total of 123 QTLs were detected for these nine floral traits of chrysanthemum, and 1069 markers were associated with them. Among these QTLs, the PVEs of 12 QTLs were over 10%, and 139 markers were associated with them. The number of QTLs that could be detected in the two study years (2015/2016) was 92, and 972 markers were associated with them (Supplementary Table 2). A total of 12 major QTLs were found for these 12 traits with PVEs >10%.

#### CTMD

**Fig. 2:**
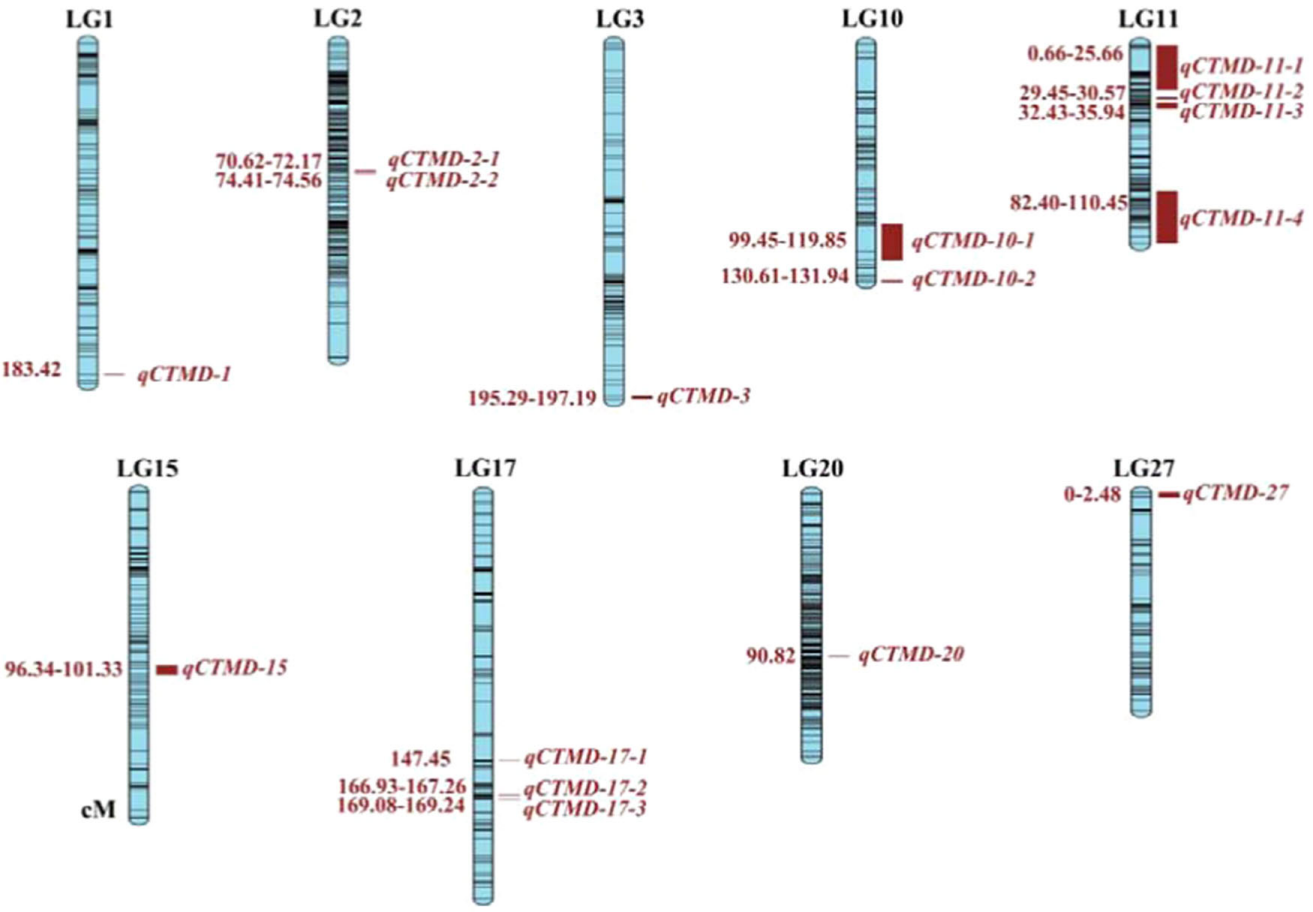
The results of the QTL test for the corolla tube merged degree. The left side of each linkage group shows the location of the QTL in units of cM. The right side shows the QTL name. This study used rice QTL nomenclature, as follows: “q” + “target traits (capital letter)” + “−” + “chromosome number or linkage group code” + “QTL number”. The full name of the QTL is usually presented in italics. CTMD Corolla tube merged degree

A total of 16 QTLs were detected for CTMD (CTL/RFL) in LG1 (183.42 cM), LG2 (70.62–74.56 cM), LG3 (195.29–197.19 cM), LG10 (99.45–131.94 cM), LG11 (0.66–35.94, 81.40–110.45 cM), LG15 (96.34–101.33 cM), LG17 (147.45–169.24 cM), LG20 (90.82 cM) and LG27 (0–2.48 cM). There were 193 markers that were closely linked to these QTLs. The LOD value ranged from 3.00 to 5.57, which explained 4.50–39.40% of the genotypic variation. Four QTLs were detected in LG11, three QTLs were detected in LG17, two QTLs were detected in LG2 and LG10, and only one QTL was detected in the other linkage groups (Fig. 2 and Supplementary Table 2).

**Fig. 3:**
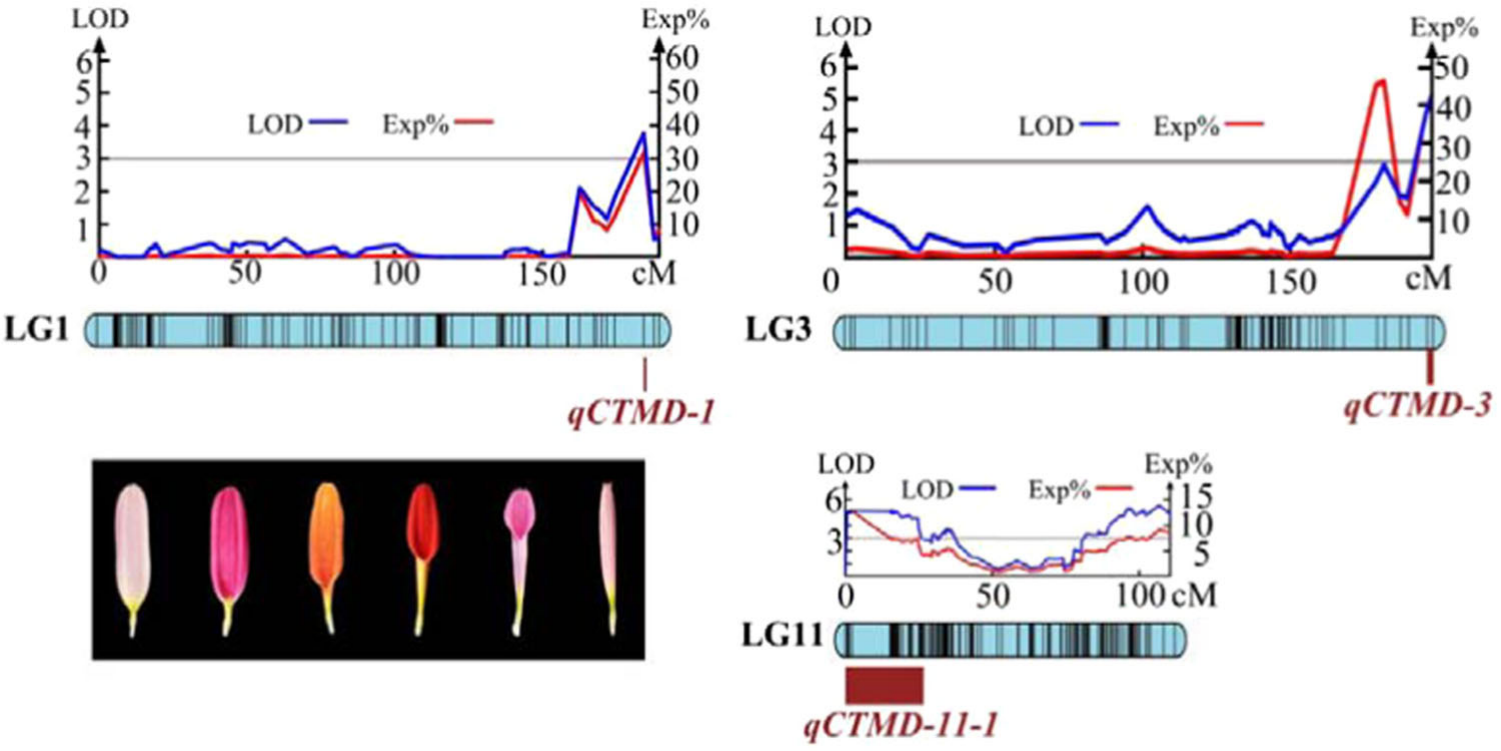
The results of the major QTL test for the corolla tube merged degree. CTMD Corolla tube merged degree

For CTMD, we found three major QTLs in LG1 (183.42 cM), LG3 (195.29–197.19 cM) and LG11 (0.66–25.26 cM). Their LOD values were 3.77, 5.09 and 5.23, which explained 31.90%, 39.40% and 13.10% of the observed genotypic variation, respectively (Fig. 3). Their flanking molecular markers were Marker224635, Marker222252 and Marker161788 (Marker161783 and Marker161785).

#### RNRF

**Fig. 4:**
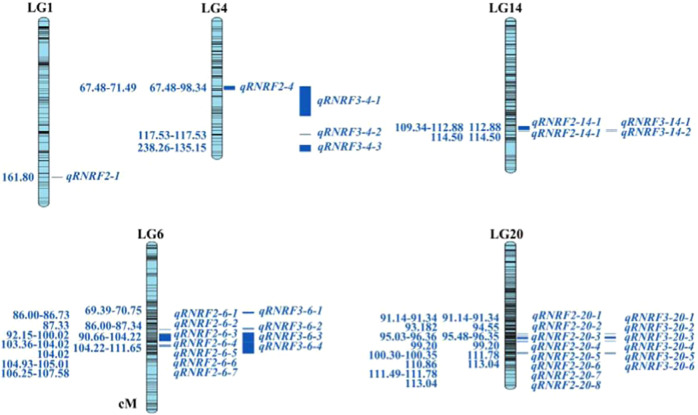
QTL test results for the relative number of ray florets. Note: RNRF2 indicates the number of ray florets/the total number of florets; RNRF3 indicates the number of whorls of ray florets/number of whorls of total florets. The locations of the QTLs are listed to the left of each linkage group in units of cM. The right side lists the QTL names

For CDFD/ID, we found one QTL in LG11 (84.825–85.719 cM). There were four markers closely linked to this QTL. The LOD value was 5.54, which explained 8.40% of the observed genotypic variation (Fig. 4 and Supplementary Table 2).

For NRF/NF, we found 19 QTLs in LG1 (161.80 cM), LG4 (67.48–71.49 cM), LG6 (86.00–107.58 cM), LG14 (109.34–114.50 cM) and LG20 (91.14–113.04 cM). There were 97 markers closely linked to these QTLs. The LOD value ranged from 3.00 to 5.22, which explained 4.40–64.60% of the genotypic variation. Among these QTLs, eight were detected in LG20; seven were detected in LG6; and one, one and two were detected in LG1, LG4 and LG14, respectively (Fig. 4 and Supplementary Table 2).

For NWRF/NWF, we found 15 QTLs in LG4 (67.48–135.15 cM), LG6 (69.39–111.65 cM), LG14 (112.88–114.50 cM) and LG20 (91.14–113.04 cM). There were 170 markers closely linked to these QTLs. The LOD value ranged from 3.05 to 4.47, which explained 4.50–11.30% of the genotypic variation. Among these QTLs, 6 were detected in LG20, and 4, 3 and 2 were detected in LG6, LG4 and LG14, respectively (Fig. 4 and Supplementary Table 2).

**Fig. 5:**
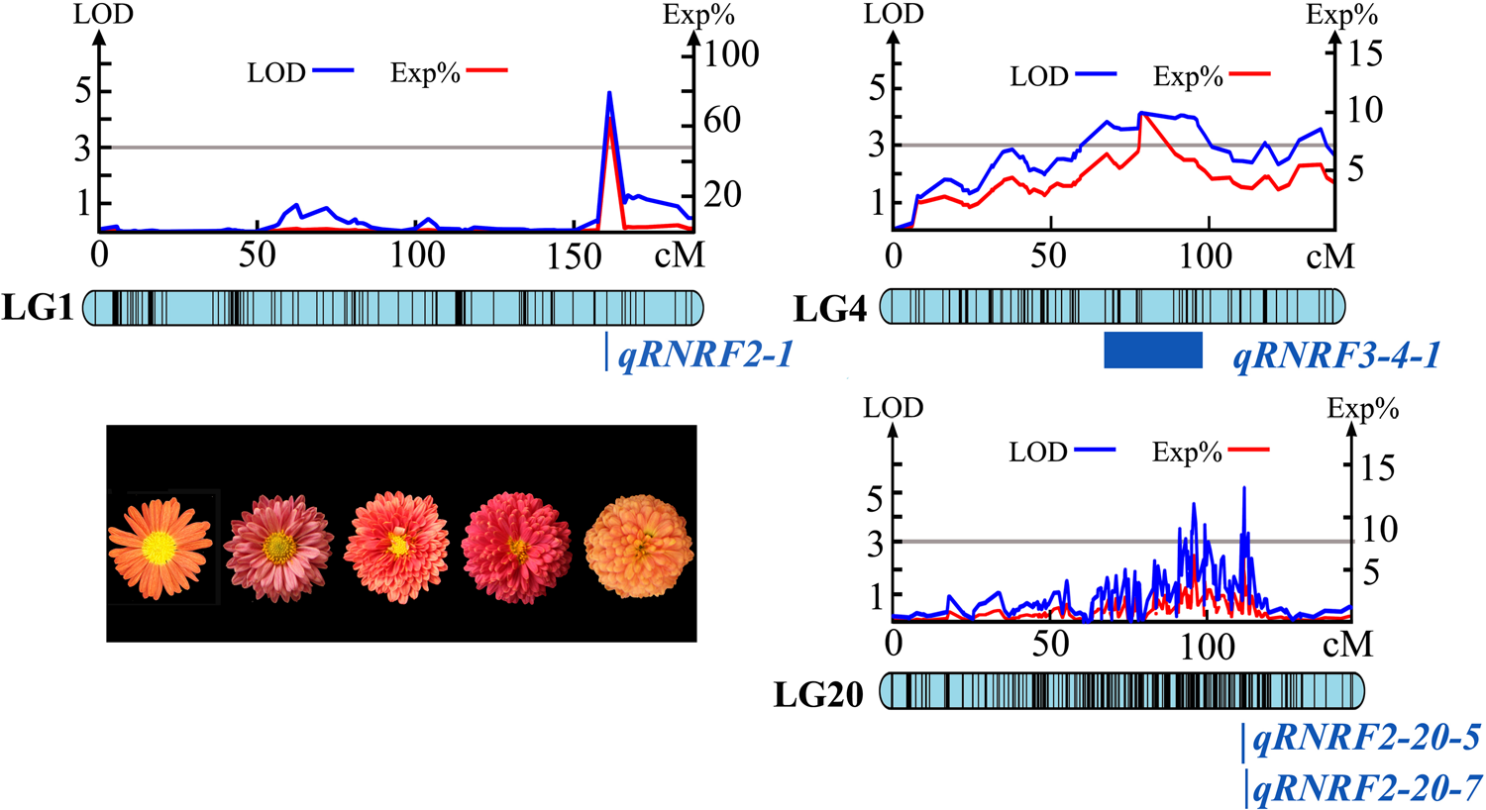
Major QTL test results for the relative number of ray florets. Note: RNRF2 is the number of ray florets/the total number of florets (NRF/NF); RNRF3 is the number of whorls of ray florets/the number of whorls of total florets (NWRF/NWF)

For NRF/NF, we found 2 major QTLs in LG1 (161.80 cM) and LG20 (111.49–111.78 cM). Their LOD values were 4.96 and 5.22, which explained 64.60% and 13.10% of the genotypic variation, respectively (Fig. 5). Their flanking molecular markers were Marker64856, Marker10768 and Marker10759. For NWRF/NWF, we found 2 major QTLs in LG4 (67.48–98.33 cM) and LG20 (111.78–111.94 cM). Their LOD values were 4.11 and 4.74, which explained 10.10% and 11.30% of the genotypic variation, respectively (Fig. 5). Their flanking molecular markers were Marker59837, Marker10768 and Marker10759. In conclusion, a total of 34 QTLs controlling RNRF and 4 major QTLs were detected.

### Synteny analysis and an attempt at candidate gene prediction

To further screen possible candidate genes underlying the detected QTLs, we aligned our genetic map with the available Asteraceae reference genomes of sunflower and lettuce since a reference genome of hexaploid chrysanthemum has not yet been published. Transcriptome unigene data from wild-type *Chrysanthemum lavandulifolium* and wild-type *Chrysanthemum vestitum* were also used as aligning pools. We attempted to screen for possible candidate genes according to macroscopic collinearity and conservation among species.

Collinearity analysis between the genetic map and the sunflower reference genome revealed that 150 of the total 6452 SNP markers could be mapped to the sunflower reference genome (Supplementary Fig. 2). Within the QTL intervals of both traits (LOD > 2), a total of 98 markers could be anchored to the sunflower reference genome. The related genes and the annotation information for the QTLs corresponding to different traits are displayed in Supplementary Table 3. Among all traits, three corresponding genes in Ha4, Ha6 and Ha17 were found to be located within the QTL for CTMD, whereas five genes in Ha8, Ha10, Ha10, Ha11 and Ha12 were in the QTL responsible for RNRF. The annotation information suggests that these genes play important roles in the membrane composition, chloroplast metabolism and redox reactions. The other traits exhibit different corresponding genes.

The same mapping approach was used between the genetic map and the lettuce genome, and it was found that 0.12% of the genetic markers could be anchored, indicating that chrysanthemum may present better collinearity with the sunflower reference genome and that similar collinearity levels exist among these three species. We focused on the comparison of the CTMD- and RNRF-related QTLs with the lettuce genome. In the CTMD trait QTL interval, 18 markers were in line with 8 scaffold sequences of the lettuce genome, and 16 markers in the RNRF QTL interval were in accordance with 11 scaffold sequences of lettuce, revealing 12 and 8 genes for CTMD and RNRF, respectively (Supplementary Table 4).

To understand the genetic relationship between *C.* × *morifolium* Ramat. and *C. seticuspe* at the genome scale, we mapped our SLAF sequencing data to the genome sequences of *C. seticuspe*^30^. We found that the average mapping rate was 73.71% (Supplementary Table 5), which was an empirically lower mapping rate than those found in these closely related species (mapping rate ≥ 90%), indicating a relatively long genetic distance from *C.* × *morifolium* Ramat. To further investigate the syntenic relationship between them, we compared the collinearity between SNP markers in our high-density genetic map and the scaffolds of the *C. seticuspe’s* genome. Only 109 out of 354,212 scaffolds (0.03%) showed synteny (Supplementary Table 6). This low degree ratio of synteny also indicated that the genetic distance between the cultivars is long, although this result can be partly attributed to the fragmented genome of *C. seticuspe*.

Furthermore, we mapped the SLAFs in the QTL regions of CTMD and RNRF to the transcriptome unigene pools of wild-type chamomile and wild-type *C. sinense*, respectively. A total of 9 unigenes were probed in wild-type chamomile and 10 unigenes in wild-type *C. sinense*. This gene information represents an attempt at producing a library for future candidate gene screening.

## Discussion

### Development of a high-quality and high-density genetic map for chrysanthemum based on SLAF-seq

The number and distribution of markers in the genome determine the uniformity and coverage of the markers in the genetic map. The markers developed from the genome are more evenly distributed and exhibit wider coverage than the markers developed from transcriptome sequences^31^. Based on SLAF-seq technology, this study developed 6452 high-quality SNP markers from the genome for the construction of a high-density genetic maps for chrysanthemum. The constructed genetic maps present high quality and meet the requirements for QTL fine mapping. The genotyping data for the 6452 markers in the mapping population were tested with the *χ*^2^ test (*P* < 0.05). The results showed that there were only 525 markers of segregation distortion, and the segregation distortion ratio was 8.14%. SLAF-seq technology can be successfully applied for the development of high-throughput SNP markers for polyploid species such as chrysanthemum. Van Geest et al.^27^ used RNA-seq to develop 30,312 SNP markers from 13,902 contigs in two parents and 405 hybrids for the construction of a high-density genetic map for chrysanthemum. Although the number of markers developed via RNA-seq is larger, the distribution of these markers is too concentrated, affecting the QTL mapping accuracy of the genetic map. Moreover, library construction based on RNA-seq technology is relatively cumbersome, especially for large groups. The KASP genotyping test also confirmed the accuracy of the markers developed in our study.

### Inheritance mode in chrysanthemum

The mode of inheritance in chrysanthemum has been controversial thus far. Cytological studies of chrysanthemums indicate that they are mostly hexaploid (2*n* = 6× = 54) and that some are aneuploid (2*n* = 36–75)^32^. At present, most chrysanthemum researchers believe that chrysanthemum originated from interspecific hybridization, with a heterozygous origin. Therefore, it is thought that chrysanthemum is allohexaploid. Moreover, by observing chromosome pairing in meiotic metaphase, the mode of inheritance was found to be disomic^24,33,34^. Many scholars have also inferred that chrysanthemum presents disomic inheritance on the basis of molecular markers^35^. However, some studies have indicated that chrysanthemum may be allohexaploid^36–38^. Van Geest et al.^37^ found that the chrysanthemum population that they studied tended to exhibit hexasomic inheritance, and these researchers constructed a high-density genetic map for chrysanthemum, containing 30,312 SNP markers and 9 linkage groups. In our study, the separation ratio of SNP markers in hybrids was calculated according to the method of Langton^39^, and the PT test was carried out for different separation ratios. It was found that the separation ratio was 1:1 in 93.7% of cases, which indicated that the mapping populations were more likely to exhibit disomic inheritance. The population examined in this study was more inclined to exhibit allohexaploidy. Based on this study, a high-density genetic map of the chrysanthemum was constructed, which contained 6452 SNP markers and 27 linkage groups.

### QTL analysis of flower traits in chrysanthemum

The flower head type of chrysanthemum is determined by many factors, such as the shape, number, relative length, and orientation of florets. In the past, only two qualitative characteristics (petal type and flower doubleness) were used to describe such complex flower head types. Therefore, due to inconsistent definition criteria or measurement errors, there are many differences in the results of genetic analyses of chrysanthemum capitulum type^3^. Fortunately, we have identified and defined two key quantitative traits that affect capitulum type: the CTMD and the RNRF^5^. To further analyze these complex flower traits, we performed a QTL analysis and extracted 16 QTLs and three major QTLs related to CTMD and 19 QTLs and 4 major QTLs related to RNRF. Previous studies have only identified QTLs related to the number of ray florets, resistance, flower color, flowering time, and disk floret degreening^26,27,40^. The results of this study provide a foundation for the screening of flower-related candidate genes and gene cloning in the future.

Previous studies have suggested that the petal type (the posture of ray florets) and flower doubleness are quality-related traits in chrysanthemum. For example, some researchers have suggested that the petal type of Compositae species shows a change from bilateral asymmetry (flat type) to radial symmetry (tubular type). Therefore, researchers have focused on genes related to floral symmetry, such as the *CYC-like* gene^41,42^. However, our study suggests that many intermediate types exist between the flat and tubular types in chrysanthemum, which means that the variation in petal type (redefined in our study as CTMD) is partly determined by floral symmetry. Because these traits are quantitative, there are still many unknown but very important genes controlling these traits that have not yet been discovered. This will limit the breeding of different chrysanthemum flower types. According to our results, the markers identified among major QTLs for CTMD and RNFR may become important tightly linked markers of CTMD and RNRF. These markers can be developed for use in the molecular marker-assisted (MAS) breeding of CTMD and RNRF in chrysanthemum. Moreover, it is possible to pyramid multiple related QTLs for flower-type breeding in the future. This will provide technical guidance for the breeding of chrysanthemum varieties with high ornamental value related to flower types (such as full-double or pompom forms).

### Future research strategy

KASP markers are often designed to determine the uniqueness of a primer in the reference genome^43–45^. For species without a reference genome, this step cannot be performed. Ten of the 16 markers reported in this article can be verified, which means that KASP markers can be used in species without an available reference genome. However, this does not mean that our SLAF markers are not sufficiently accurate. Conversely, for species without a reference genome, the conversion of SNP markers developed using SLAF-seq to KASP markers can still be considered for rapid large-population genotyping.

In addition, we screened a sample of genes by comparison with the sunflower and lettuce reference genomes and two wild chrysanthemum transcriptomes. Using these genes, we can design primers for fluorescent quantitative PCR for preliminary verification. If the QTL interval is large and the verification effect is not satisfactory, we can continue to construct an F_1_ population or a BC population and use the QTL interval markers designed on the basis of KASP genotyping primers to narrow the QTL intervals. The comparison of our chrysanthemum genetic map with several reference genomes belonging to Asteraceae revealed collinear segments, and the obtained sequence information can guide the development of new markers. Based on this study, we believe that individual important genes that are contained within these important QTLs and their functions will be revealed in the near future.

## Materials and methods

### Mapping population construction and sampling

**Fig. 6:**
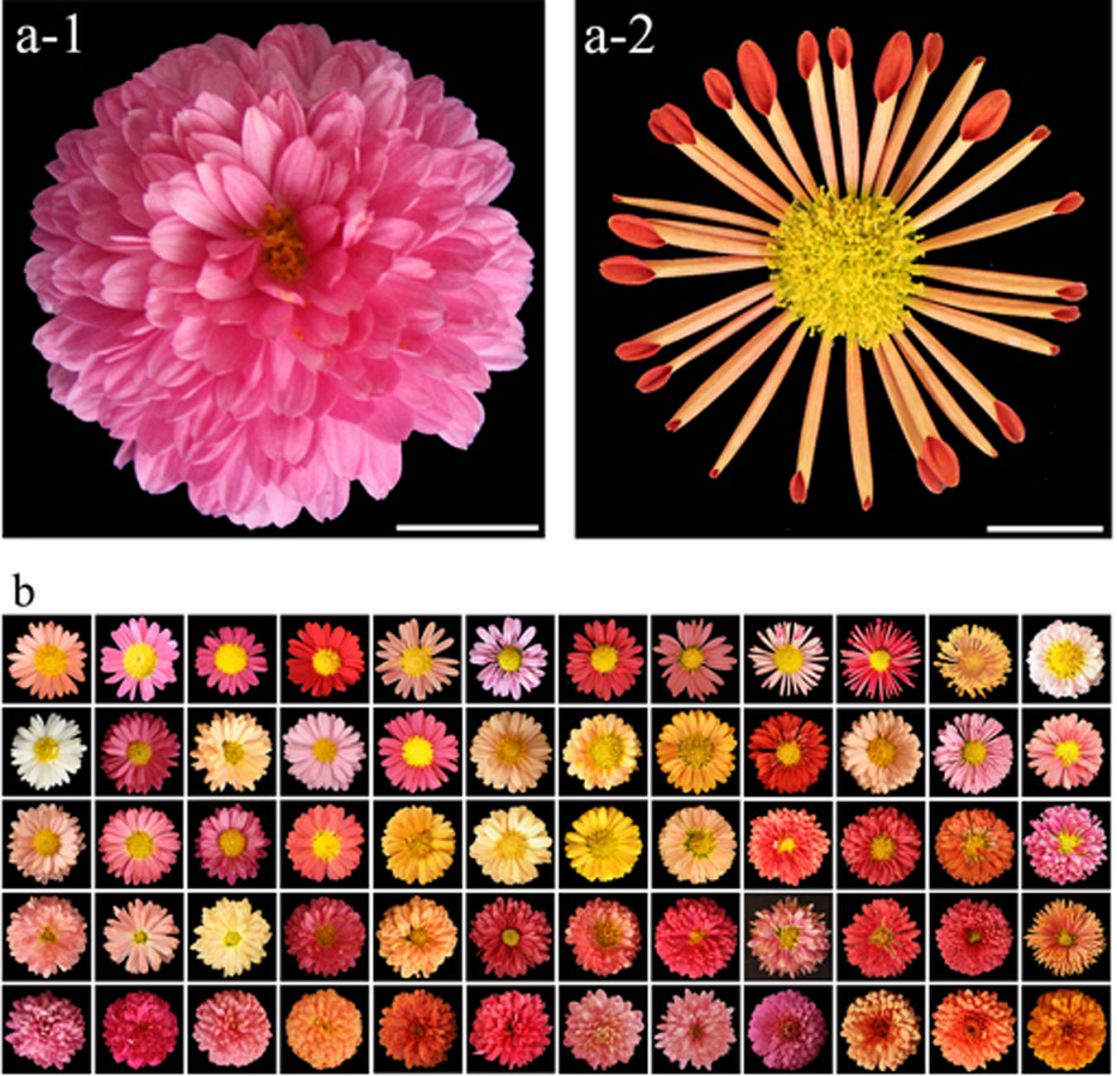
The pair of chrysanthemum parents (a) and some flowers from of the F1 population (b). Note: **a-1** female parent ‘Candy’; **a-2** male parent ‘225’; **b** a selection of the hybrid offspring with the differences in CTMD and the relative number of ray florets. Scale is 1 cm

An F_1_ mapping population containing 305 individuals was obtained from an artificial cross between *Chrysanthemum* × *morifolium* ‘Candy’ and *C*. × *morifolium* ‘225’. The female parent ‘Candy’ exhibited double-type and flat-type flowers (Fig. 6a-1), whereas the male parent ‘225’ showed single-type and tubular-type flowers (Fig. 6a-2). The flower traits of the parents and hybrids showed stable performance after at least 2 years of observations^5^. These two parents were suggested to be hexaploid on the basis of karyotyping and flow cytometry (Fig. 6, Supplementary Figs. 3 and 4). Crossing by hand pollination was performed to generate F_1_ seeds during the autumn of 2013. The F_1_ hybrid seeds were directly sown in May 2014, and their branches were then used to propagate three individual plants for every hybrid based on cutting slips with a spacing of 50 cm. During the growth period, standard protocols for water and fertilizer application and disease and insect pest control were adopted^5^. A total of 13 characteristics were investigated in the parents and all F_1_ hybrids during flowering periods in the autumns of 2015 and 2016 (Table 4). The materials used for the experiment were all stored at the Nursery of Beijing Forestry University, Beijing, China (40.1608°N, 116.4595°E).

**Table 4:** Thirteen floral morphological characteristics and the corresponding measurement

No.	Traits	Abb.
C1	Inflorescence diameter/cm	ID
C2	Center disc flower diameter/cm	CDFD
C3	Center disc flower diameter/inflorescence diameter	CDFD/ID
C4	Number of whorls of ray florets	NWRF
C5	Number of whorls of disc florets	NWDF
C6	Number of whorls of ray florets/number of whorls of florets	NWRF/NWF
C7	Number of ray florets	NRF
C8	Number of disc florets	NDF
C9	Number of ray florets/number of florets	NRF/NF
C10	Ray floret length/cm	RFL
C11	Corolla tube length/cm	CTL
C12	Corolla tube length/ray floret length (corolla tube merged degree)	CTL/RFL (CTMD)
C13	Ray floret width	RFW

Total DNA was extracted from fresh young leaves of the above two parents and 305 F_1_ individuals separately using an Extraction Kit for Plant Genomic DNA (Tiangen Biotech Co., Beijing, China). The quality of the genomic DNA was controlled by electrophoresis in a 1% agarose gel with standard lambda CK DNA and an ND-2000 spectrophotometer (NanoDrop, Wilmington, DE, USA). DNA samples were stored at −20 °C for later specific-locus amplified fragment sequencing (SLAF) library construction.

### Phenotyping of the mapping population

**Fig. 7:**
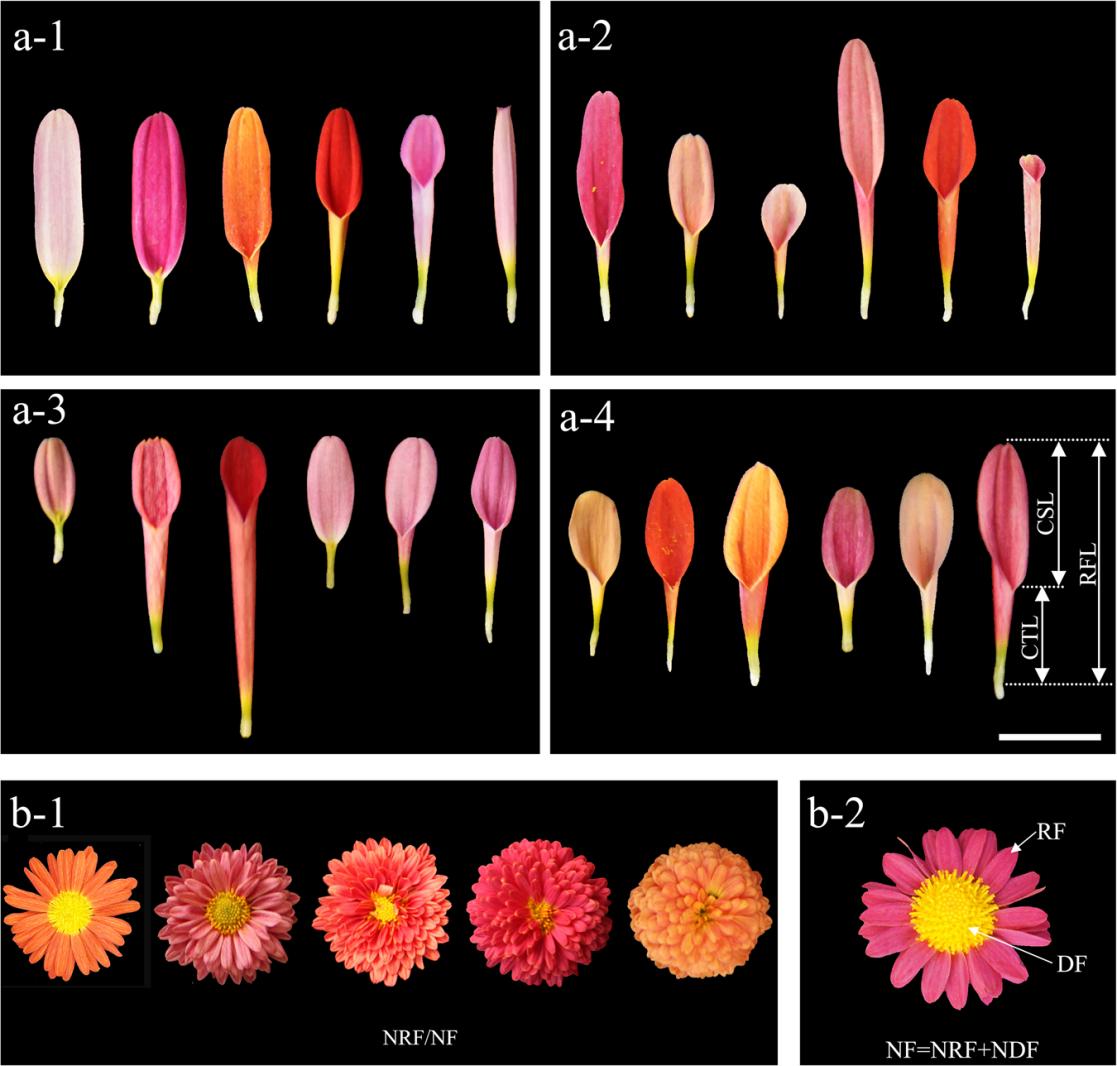
Morphological variation of ray florets in hybrids. Note: Only RFL is the same (**a**-1); only CTL is the same (**a**-2); only CSL is the same (**a**-3); CTL/RFL is the same (**a**-4). Ray floret length (RFL); corolla tube length (CTL); corolla splitting length (CSL). Morphological variation of flower heads with different numbers of florets (**b**-1). The scale bar represents 0.5 cm

A total of 13 flower characteristics of the parents and F_1_ hybrids were investigated during flowering periods in the autumns of 2015 and 2016 (Table 4). Based on our previous research, we believe that CTMD and RNRF are the two most critical traits that determine the chrysanthemum capitulum type^5^. In this study, the ratio of CTL to RFL (CTL/RFL) was used to describe CTMD (Fig. 7a). The ratio of NRF to NDF (NRF/NDF) (Table 4) was used to describe RNRF. The ratio of NWRF to the number of whorls in florets (NWRF/NWF) as well as the CDFD/ID ratio can also be used to describe RNRF (Fig. 7b). The detailed measurement methods and the analysis of phenotypic traits are described in Song et al.^2^.

### SLAF library construction, Illumina sequencing and data preparation

*Hae*III (New England Biolabs, NEB, USA) was used to perform an enzyme digestion test on the genomic DNA from each sample. The digested fragment was then subjected to 3′ end plus A (nucleotide) treatment, and a dual-index (PAGE-purified, Life Technologies, USA) sequencing linker was ligated to the A-tailed fragment, followed by PCR amplification, as referenced by Luo et al.^46^. The target fragment (414–514 bp) was amplified from the DNA obtained by gel recovery, and sequencing was performed using the Illumina HiSeq^TM^ 2500 platform (Illumina, Inc; San Diego, CA, US) according to the manufacturer’s recommendations. To monitor the accuracy of library construction and the sequencing test, the same protocol was followed for the genomic DNA of *Oryza sativa* L. *japonica* (http://rapdb.dna.affrc.go.jp/) as a control trial. The resulting sequencing data were processed with an in-house Perl script for quality control. Reads that occupied >50% of bases with Q values ≤ 10 or a ratio of ambiguous sequence content (“*N*”) > 10% were discarded. The remaining reads were individually sorted using duplex barcode sequences. To ensure the quality of sequencing, the percentage of bases whose sequencing quality value was ≥30 was calculated in relation to the total bases (Q30 percentage), and the percentage of G and C bases among the total number of bases in the sequencing results (GC percentage) was also calculated. All clean sequencing data were uploaded to the SRA (sequence read archive) database of NCBI.

### SNP marker development and genotyping

The sorted clean sequences from each sample were clustered according to sequence similarity (identity = 90%) by using BLAT with default parameters^47^. Single nucleotide polymorphisms (SNPs) were called between the parents and the F_1_ individuals for each cluster of SLAF loci, and SLAFs with more than five SNPs were excluded. In the mapping populations of diploid chrysanthemums, one locus cannot exhibit more than four different SLAF genotypes, so clusters with more than four tags were treated as repetitive SLAFs and excluded from later analysis. The polymorphic SLAF markers in each parent showing a depth below 10-fold were also excluded. The remaining polymorphic SLAFs were then coded according to aa×bb, ab×cd, ab×cc, cc×ab, ef×eg, hk×hk, lm×ll and nn×np segregation patterns, and all patterns except for aa×bb were deemed suitable for the CP (cross-pollinator) population type.

### Construction of the genetic linkage map

To construct a high-quality genetic map, polymorphic SNP markers showing integrity scores ≤70% (markers that could be genotyped in ≤70% of F_1_ individuals) and significant segregation distortion (*P* < 0.01) according to the *χ*^2^ test were initially (*P* < 0.01) excluded. Finally, all high-quality SNP markers were grouped with a single-linkage clustering algorithm with the following settings: logarithm of odds (LOD) ≥ 5 and a maximum recombination rate of 0.4. HighMap software^48^ was used to order the SNP markers and correct genotyping errors. Genetic map distance was calculated using the Kosambi function.

### Data analysis and QTL mapping

QTL analysis of 13 flower traits was performed using MapQTL® 6 software^49^. First, the threshold of the logarithm of odds (LOD) scores for evaluating the statistical significance (*P* < 0.05) of QTL effects was determined using 1000 permutations. For some traits that did not reach a significant LOD threshold, we manually adjusted the LOD to 3. Then, we scanned the QTLs in every 1 cM interval in each linkage group and calculated their contribution rate by using the interval mapping (IM) model.

### Kompetitive Allele-Specific PCR (KASP) trials

A set of 15 SLAFs containing SNPs for which primers could be designed identified from the QTLs for CTMD were selected to run the KASP trials; all primers were designed with Primer 5.0 and are shown in Supplementary Table 5. We randomly selected 80 individuals from the F_1_ population for KASP genotyping (Supplementary Table 6). A 1536-well plate was used to perform KASP according to the protocol provided by LGC Genomics (LGC, Middlesex, UK). PCR (Bio-Rad, Hercules, CA) was performed using the following program: thermal activation at 95 °C for 16 min, denaturation at 95 °C for 20 s, primer annealing at 65 °C for 55 s (decreasing by 1 °C per cycle, 10 cycles total) and, finally, 30 cycles of amplification (95 °C for 10 s; 57 °C for 60 s). A Synergy H1 full-function microplate reader (FLUO star Omega, BMG Labtech, Germany) was used to read the fluorescence signal upon the completion of the reaction.

### Synteny analysis and candidate gene prediction

The SNP markers in our high-density genetic map were anchored to the genome sequences of sunflower (https://www.ebi.ac.uk/ena/data/search?query=MNCJ00000000) and lettuce (https://www.ncbi.nlm.nih.gov/bioproject/PRJNA173551) by using BLAST+(ftp://ftp.ncbi.nlm.nih.gov/blast/executables/blast+/LATEST) with the parameters of an *E*-value < 1E−5 and bitscore >60. The final position was defined by the longest mapped sequences aligned to the reference genome sequences of sunflower and lettuce. The BLAST method applied for the lettuce genome was the same as that for the sunflower genome. However, the lettuce genome has not yet been mapped to chromosomes, so only some candidate scaffold sequences have been screened. The results were visualized by using the Circos tool (http://circos.ca)^50^. Candidate genes (or candidate scaffold sequences) in QTL regions associated with CTMD and RNRF were extrapolated based on *H. annuus* L. (2× = 2*n* = 34, https://sunflowergenome.org/index.html) and *Lactuca sativa* (2× = 2*n* = 18, http://lgr.genomecenter.ucdavis.edu/) genome annotation information. In addition, SNP makers in some major QTL regions were aligned to transcriptome sequences via the same method. Candidate genes within the QTL region were screened based on enrichment analysis of the annotated genes and the unigene hits obtained by GO and KEGG analyses.

## Supplementary information

Supplementary Table 1

Supplementary Table 2

Supplementary Table 3

Supplementary Table 4

Supplementary Table 5

Supplementary Table 6

Supplementary Table 7

Supplementary Table 8

Supplementary Figure 1

Supplementary Figure 2

Supplementary Figure 3

Supplementary Figure 4
