# Supplementary material for: High-density genetic map construction and identification of loci controlling flower-type traits in Chrysanthemum (Chrysanthemum × morifolium Ramat.)
Source: Hortic Res. 2020 Jul 1;7:108. doi: 10.1038/s41438-020-0333-1 (PMC7326996; doi:10.1038/s41438-020-0333-1)
Supplement: Supplementary file 1 — Supplementary Table 1 [file 41438_2020_333_MOESM1_ESM.docx]

**Supplementary Table 1-1** Description on basic characteristics of the 27 linkage groups of female

| Linkage  group | Total marker | Total distance(cM) | Average distance(cM) | Gap<5 cM（%） | Max Gap(cM) |
| --- | --- | --- | --- | --- | --- |
| LG1 | 3 | 0 | 0 | 100 | 0 |
| LG2 | 329 | 177.12 | 0.54 | 98.48 | 10.51 |
| LG3 | 124 | 198.91 | 1.6 | 89.43 | 15.21 |
| LG4 | 67 | 121.4 | 1.81 | 90.91 | 20.36 |
| LG5 | 351 | 194.21 | 0.55 | 98.86 | 7.38 |
| LG6 | 82 | 111.91 | 1.36 | 96.3 | 14.77 |
| LG7 | 143 | 207.64 | 1.45 | 90.85 | 19.87 |
| LG8 | 145 | 208.73 | 1.44 | 92.36 | 45.41 |
| LG9 | 124 | 250.5 | 2.02 | 95.93 | 91.56 |
| LG10 | 82 | 95.75 | 1.17 | 93.83 | 22.37 |
| LG11 | 65 | 92.89 | 1.43 | 90.62 | 17.02 |
| LG12 | 108 | 104.29 | 0.97 | 92.52 | 13.04 |
| LG13 | 160 | 266.7 | 1.67 | 92.45 | 52.37 |
| LG14 | 105 | 290.86 | 2.77 | 78.85 | 23.4 |
| LG15 | 114 | 156.56 | 1.37 | 94.69 | 47.06 |
| LG16 | 19 | 24.33 | 1.28 | 94.44 | 17.02 |
| LG17 | 115 | 214.78 | 1.87 | 88.6 | 54.28 |
| LG18 | 99 | 84.66 | 0.86 | 95.92 | 16.56 |
| LG19 | 130 | 101.37 | 0.78 | 96.12 | 20.35 |
| LG20 | 204 | 142.27 | 0.7 | 98.03 | 8.89 |
| LG21 | 102 | 92.49 | 0.91 | 94.06 | 18.42 |
| LG22 | 87 | 108.64 | 1.25 | 93.02 | 24.46 |
| LG23 | 128 | 122.8 | 0.96 | 96.06 | 12.61 |
| LG24 | 4 | 3.02 | 0.76 | 100 | 1.61 |
| LG25 | 103 | 110.26 | 1.07 | 94.12 | 17.48 |
| LG26 | 126 | 158.32 | 1.26 | 92.8 | 26.09 |
| LG27 | 99 | 118.51 | 1.2 | 91.84 | 14.33 |
| Total | 3,218 | 3,758.92 | 1.16 | 94.23 | 91.56 |

**Supplementary Table 1-2** Description on basic characteristics of the 27 linkage groups of male

| Linkage  group | Total marker | Total distance(cM) | Average distance(cM) | Gap<5 cM（%） | Max Gap(cM) |
| --- | --- | --- | --- | --- | --- |
| LG1 | 136 | 193.74 | 1.42 | 91.11 | 14.72 |
| LG2 | 208 | 92.37 | 0.44 | 99.52 | 6.04 |
| LG3 | 2 | 0.17 | 0.09 | 100 | 0.17 |
| LG4 | 75 | 139.37 | 1.86 | 83.78 | 10.14 |
| LG5 | 94 | 364.86 | 3.88 | 80.65 | 52.26 |
| LG6 | 227 | 181.81 | 0.8 | 96.9 | 38.58 |
| LG7 | 113 | 204.06 | 1.81 | 92.86 | 75.78 |
| LG8 | 44 | 4.96 | 0.11 | 100 | 0.99 |
| LG9 | 202 | 270.53 | 1.34 | 93.53 | 27.21 |
| LG10 | 57 | 131.94 | 2.31 | 85.71 | 25 |
| LG11 | 149 | 104.95 | 0.7 | 98.65 | 5.91 |
| LG12 | 150 | 172.26 | 1.15 | 93.29 | 15.66 |
| LG13 | 364 | 234.52 | 0.64 | 97.52 | 16.96 |
| LG14 | 151 | 123.9 | 0.82 | 96.67 | 7.72 |
| LG15 | 112 | 174.17 | 1.56 | 93.69 | 43.02 |
| LG16 | 170 | 133.48 | 0.79 | 97.04 | 10.95 |
| LG17 | 145 | 183.07 | 1.26 | 92.36 | 46.23 |
| LG18 | 52 | 39.19 | 0.75 | 96.08 | 14.1 |
| LG19 | 50 | 70 | 1.4 | 93.88 | 28.93 |
| LG20 | 275 | 135.61 | 0.49 | 98.91 | 7.42 |
| LG21 | 122 | 146.28 | 1.2 | 95.87 | 35.15 |
| LG22 | 81 | 126.11 | 1.56 | 93.75 | 28.93 |
| LG23 | 2 | 0 | 0 | 100 | 0 |
| LG24 | 151 | 115.38 | 0.76 | 96.67 | 8.95 |
| LG25 | 138 | 140.43 | 1.02 | 94.16 | 13.04 |
| LG26 | 78 | 89.41 | 1.15 | 94.81 | 21.35 |
| LG27 | 62 | 120.66 | 1.95 | 86.89 | 32.57 |
| Total | 3,410 | 3,693.23 | 1.16 | 94.23 | 75.78 |
