# Supplementary material for: High-density genetic map construction and identification of loci controlling flower-type traits in Chrysanthemum (Chrysanthemum × morifolium Ramat.)
Source: Hortic Res. 2020 Jul 1;7:108. doi: 10.1038/s41438-020-0333-1 (PMC7326996; doi:10.1038/s41438-020-0333-1)
Supplement: Supplementary file 2 — Supplementary Table 2 [file 41438_2020_333_MOESM2_ESM.docx]

**Supplementary Table 2** The results of QTLs test for 14 quantitative traits

of flower in chrysanthemum

| Traits | QTL code | Linkage group | The number of QTLs | The number of QTL associated markers | The position on the genetic map | LOD threshold | Exp% |
| --- | --- | --- | --- | --- | --- | --- | --- |
| ID | *qID-11-1* | LG11 | 2 | 19 | 76.63-78.12 | 6.69 | 9.60 |
|  | *qID-11-2* | LG11 |  |  | 80.51-85.72 | 6.96 | 10.20 |
| CDFD | *qCDFD-11* | LG11 | 1 | 1 | 85.72-85.72 | 5.81 | 8.80 |
| RNRF (CDFD/ID) | *qRNRF1-11* | LG11 | 1 | 4 | 84.83-85.72 | 5.54 | 8.40 |
| NWDF | *qNWDF-11* | LG11 | 10 | 30 | 80.51-85.72 | 3.26 | 5.60 |
|  | *qNWDF-14* | LG14 |  |  | 112.88-112.88 | 3.13 | 4.70 |
|  | *qNWDF-20-1* | LG20 |  |  | 83.28-83.28 | 3.05 | 7.00 |
|  | *qNWDF-20-2* | LG20 |  |  | 91.27-91.27 | 3.04 | 4.60 |
|  | *qNWDF-20-3* | LG20 |  |  | 95.75-96.35 | 4.19 | 7.10 |
|  | *qNWDF-20-4* | LG20 |  |  | 99.06-99.06 | 3.65 | 5.60 |
|  | *qNWDF-20-5* | LG20 |  |  | 99.2-99.2 | 3.02 | 4.60 |
|  | *qNWDF-20-6* | LG20 |  |  | 110.86-110.86 | 3.34 | 5.00 |
|  | *qNWDF-20-7* | LG20 |  |  | 111.78-111.78 | 3.97 | 8.90 |
|  | *qNWDF-20-8* | LG20 |  |  | 113.04-113.04 | 3.00 | 4.60 |
| NWRF | *qNWRF-4-1* | LG4 | 12 | 163 | 57.03-57.03 | 3.01 | 4.50 |
|  | *qNWRF-4-2* | LG4 |  |  | 58.02-58.02 | 3.00 | 4.50 |
|  | *qNWRF-4-3* | LG4 |  |  | 59.11-98.33 | 4.05 | 10.10 |
|  | *qNWRF-6-1* | LG6 |  |  | 69.39-70.75 | 3.26 | 5.00 |
|  | *qNWRF-6-2* | LG6 |  |  | 86-87.33 | 3.55 | 5.30 |
|  | *qNWRF-6-3* | LG6 |  |  | 90.66-104.02 | 4.38 | 6.50 |
|  | *qNWRF-6-4* | LG6 |  |  | 104.02-111.65 | 3.60 | 5.30 |
|  | *qNWRF-20-1* | LG20 |  |  | 91.14-91.34 | 3.19 | 4.80 |
|  | *qNWRF-20-2* | LG20 |  |  | 95.48-96.35 | 3.81 | 6.80 |
|  | *qNWRF-20-3* | LG20 |  |  | 100.95-100.95 | 3.02 | 5.20 |
|  | *qNWRF-20-4* | LG20 |  |  | 111.78-111.78 | 4.05 | 9.10 |
|  | *qNWRF-20-5* | LG20 |  |  | 113.04-113.04 | 3.09 | 4.70 |
| RNRF | *qRNRF3-4-1* | LG4 | 15 | 170 | 67.48-98.33 | 4.11 | 10.10 |
| (NWRF/NWF) | *qRNRF3-4-2* | LG4 |  |  | 117.53-117.53 | 3.06 | 4.50 |
|  | *qRNRF3-4-3* | LG4 |  |  | 128.26-135.15 | 3.54 | 5.50 |
|  | *qRNRF3-6-1* | LG6 |  |  | 69.39-70.75 | 3.05 | 4.70 |
|  | *qRNRF3-6-2* | LG6 |  |  | 86-87.33 | 3.30 | 5.00 |
|  | *qRNRF3-6-3* | LG6 |  |  | 90.66-104.02 | 4.41 | 6.50 |
|  | *qRNRF3-6-4* | LG6 |  |  | 104.02-111.65 | 3.68 | 5.40 |
|  | *qRNRF3-14-1* | LG14 |  |  | 112.88-112.88 | 3.37 | 5.10 |
|  | *qRNRF3-14-2* | LG14 |  |  | 114.5-114.5 | 3.10 | 4.60 |
|  | *qRNRF3-20-1* | LG20 |  |  | 91.14-91.34 | 3.25 | 4.90 |
|  | *qRNRF3-20-2* | LG20 |  |  | 94.55-94.55 | 3.06 | 4.60 |
|  | *qRNRF3-20-3* | LG20 |  |  | 95.48-96.35 | 4.47 | 8.10 |
|  | *qRNRF3-20-4* | LG20 |  |  | 99.2-99.2 | 3.33 | 5.10 |
|  | *qRNRF3-20-5* | LG20 |  |  | 111.78-111.94 | 4.74 | 11.30 |
|  | *qRNRF3-20-6* | LG20 |  |  | 113.04-113.04 | 3.42 | 5.20 |
| NDF | *qNDF-14* | LG14 | 1 | 1 | 112.88-112.88 | 4.73 | 7.10 |
| NRF | *qNRF-6-1* | LG6 | 12 | 74 | 86-86.73 | 3.10 | 4.60 |
|  | *qNRF-6-2* | LG6 |  |  | 92.15-100.02 | 4.06 | 6.00 |
|  | *qNRF-6-3* | LG6 |  |  | 103.36-104.02 | 3.10 | 4.90 |
|  | *qNRF-6-4* | LG6 |  |  | 106.25-106.58 | 3.04 | 4.50 |
|  | *qNRF-14-1* | LG14 |  |  | 42.6-42.6 | 3.14 | 4.80 |
|  | *qNRF-14-2* | LG14 |  |  | 49.37-49.37 | 3.06 | 4.70 |
|  | *qNRF-14-3* | LG14 |  |  | 114.5-114.5 | 3.02 | 4.50 |
|  | *qNRF-19* | LG19 |  |  | 87.91-98.77 | 3.42 | 5.10 |
|  | *qNRF-20-1* | LG20 |  |  | 91.27-91.27 | 3.09 | 4.70 |
|  | *qNRF-20-2* | LG20 |  |  | 95.48-96.35 | 3.75 | 7.50 |
|  | *qNRF-20-3* | LG20 |  |  | 111.78-111.78 | 3.16 | 7.30 |
|  | *qNRF-24* | LG24 |  |  | 116.89-116.89 | 3.02 | 8.80 |
| RNRF | *qRNRF2-1* | LG1 | 19 | 97 | 161.8-161.8 | 4.96 | 64.60 |
| (NRF/NF) | *qRNRF2-4* | LG4 |  |  | 67.48-71.49 | 3.28 | 5.50 |
|  | *qRNRF2-6-1* | LG6 |  |  | 86-86.73 | 3.20 | 4.70 |
|  | *qRNRF2-6-2* | LG6 |  |  | 87.33-87.33 | 3.00 | 4.50 |
|  | *qRNRF2-6-3* | LG6 |  |  | 92.15-100.02 | 3.98 | 5.90 |
|  | *qRNRF2-6-4* | LG6 |  |  | 103.36-104.02 | 3.12 | 4.90 |
|  | *qRNRF2-6-5* | LG6 |  |  | 104.02-104.02 | 3.12 | 4.90 |
|  | *qRNRF2-6-6* | LG6 |  |  | 104.93-105.01 | 3.00 | 4.40 |
|  | *qRNRF2-6-7* | LG6 |  |  | 106.25-107.58 | 3.02 | 4.50 |
|  | *qRNRF2-14-1* | LG14 |  |  | 109.34-112.88 | 4.24 | 6.30 |
|  | *qRNRF2-14-2* | LG14 |  |  | 114.5-114.5 | 4.15 | 6.20 |
|  | *qRNRF2-20-1* | LG20 |  |  | 91.14-91.34 | 3.55 | 5.30 |
|  | *qRNRF2-20-2* | LG20 |  |  | 93.18-93.18 | 3.30 | 5.00 |
|  | *qRNRF2-20-3* | LG20 |  |  | 95.03-96.35 | 4.50 | 9.40 |
|  | *qRNRF2-20-4* | LG20 |  |  | 99.2-99.2 | 3.70 | 5.70 |
|  | *qRNRF2-20-5* | LG20 |  |  | 100.3-100.35 | 3.05 | 4.60 |
|  | *qRNRF2-20-6* | LG20 |  |  | 110.86-110.86 | 3.32 | 5.00 |
|  | *qRNRF2-20-7* | LG20 |  |  | 111.49-111.78 | 5.22 | 13.10 |
|  | *qRNRF2-20-8* | LG20 |  |  | 113.04-113.04 | 3.38 | 5.20 |
| RFL | *qRFL-11-1* | LG11 | 22 | 218 | 21.13-21.87 | 3.13 | 4.60 |
|  | *qRFL-11-2* | LG11 |  |  | 24.52-24.52 | 3.24 | 6.90 |
|  | *qRFL-11-3* | LG11 |  |  | 29.09-29.45 | 3.14 | 6.30 |
|  | *qRFL-11-4* | LG11 |  |  | 32.43-42.26 | 4.14 | 6.20 |
|  | *qRFL-11-5* | LG11 |  |  | 73.94-93.25 | 6.33 | 9.60 |
|  | *qRFL-15* | LG15 |  |  | 161.34-161.34 | 10.61 | 22.50 |
|  | *qRFL-17* | LG17 |  |  | 42.62-56.03 | 4.40 | 6.50 |
|  | *qRFL-19-1* | LG19 |  |  | 34.04-45.45 | 4.27 | 6.30 |
|  | *qRFL-19-2* | LG19 |  |  | 45.45-55.81 | 4.80 | 8.40 |
|  | *qRFL-20-1* | LG20 |  |  | 77.05-77.27 | 4.11 | 7.00 |
|  | *qRFL-20-2* | LG20 |  |  | 77.55-77.55 | 4.25 | 7.50 |
|  | *qRFL-20-3* | LG20 |  |  | 83.28-83.28 | 3.54 | 8.20 |
|  | *qRFL-20-4* | LG20 |  |  | 84.11-84.11 | 3.10 | 6.00 |
|  | *qRFL-20-5* | LG20 |  |  | 96.7-96.7 | 3.17 | 4.80 |
|  | *qRFL-20-6* | LG20 |  |  | 101.8-101.8 | 3.12 | 4.90 |
|  | *qRFL-20-7* | LG20 |  |  | 105.14-105.2 | 3.98 | 7.80 |
|  | *qRFL-20-8* | LG20 |  |  | 105.71-105.71 | 3.03 | 6.40 |
|  | *qRFL-20-9* | LG20 |  |  | 110.15-110.41 | 4.90 | 7.40 |
|  | *qRFL-20-10* | LG20 |  |  | 110.92-111.21 | 4.35 | 6.50 |
|  | *qRFL-20-11* | LG20 |  |  | 113.38-113.38 | 4.76 | 7.20 |
|  | *qRFL-20-12* | LG20 |  |  | 115.75-116.36 | 4.23 | 6.60 |
|  | *qRFL-20-13* | LG20 |  |  | 118.89-123.48 | 3.82 | 6.00 |
| CTL | *qCTL-1* | LG1 | 12 | 99 | 173.64-183.42 | 3.00 | 19.40 |
|  | *qCTL-2-1* | LG2 |  |  | 5.1-11.46 | 3.20 | 5.30 |
|  | *qCTL-2-2* | LG2 |  |  | 70.62-70.62 | 3.01 | 5.70 |
|  | *qCTL-11-1* | LG11 |  |  | 0.66-18.23 | 3.49 | 8.90 |
|  | *qCTL-11-2* | LG11 |  |  | 21.13-21.87 | 3.07 | 4.60 |
|  | *qCTL-11-3* | LG11 |  |  | 97.14-97.14 | 3.03 | 4.50 |
|  | *qCTL-11-4* | LG11 |  |  | 100.76-101.36 | 3.12 | 4.60 |
|  | *qCTL-11-5* | LG11 |  |  | 106.71-106.71 | 3.36 | 5.50 |
|  | *qCTL-15* | LG15 |  |  | 96.34-101.33 | 3.26 | 7.20 |
|  | *qCTL-17-1* | LG17 |  |  | 133.25-148.1 | 3.71 | 8.00 |
|  | *qCTL-17-2* | LG17 |  |  | 165.27-169.24 | 3.51 | 5.20 |
|  | *qCTL-24* | LG24 |  |  | 105.8-106.13 | 3.47 | 29.20 |
| CTMD | *qCTMD-1* | LG1 | 16 | 193 | 183.42 | 3.77 | 31.90 |
| (CTL/RFL) | *qCTMD-2-1* | LG2 |  |  | 70.62-72.17 | 3.69 | 7.00 |
|  | *qCTMD-2-2* | LG2 |  |  | 74.41-74.56 | 3.25 | 6.70 |
|  | *qCTMD-3* | LG3 |  |  | 195.29-197.19 | 5.09 | 39.40 |
|  | *qCTMD-10-1* | LG10 |  |  | 99.45-119.85 | 3.49 | 6.60 |
|  | *qCTMD-10-2* | LG10 |  |  | 130.61-131.94 | 3.11 | 4.60 |
|  | *qCTMD-11-1* | LG11 |  |  | 0.66-25.26 | 5.23 | 13.10 |
|  | *qCTMD-11-2* | LG11 |  |  | 29.45-30.57 | 3.27 | 5.00 |
|  | *qCTMD-11-3* | LG11 |  |  | 32.43-35.94 | 3.79 | 5.60 |
|  | *qCTMD-11-4* | LG11 |  |  | 81.4-110.45 | 5.57 | 9.10 |
|  | *qCTMD-15* | LG15 |  |  | 96.34-101.33 | 3.37 | 7.40 |
|  | *qCTMD-17-1* | LG17 |  |  | 147.45-147.45 | 3.09 | 5.30 |
|  | *qCTMD-17-2* | LG17 |  |  | 166.93-167.26 | 3.27 | 4.90 |
|  | *qCTMD-17-3* | LG17 |  |  | 169.08-169.24 | 3.00 | 4.50 |
|  | *qCTMD-20* | LG20 |  |  | 90.82-90.82 | 3.59 | 6.60 |
|  | *qCTMD-27* | LG27 |  |  | 0-2.48 | 3.36 | 5.50 |
| RFW | *qRFW-12-1* | LG12 | 6 | 61 | 41.53-46.41 | 3.96 | 6.90 |
|  | *qRFW-12-2* | LG12 |  |  | 53.53-62.6 | 3.67 | 5.70 |
|  | *qRFW-17* | LG17 |  |  | 104.97-104.97 | 3.00 | 4.80 |
|  | *qRFW-20-1* | LG20 |  |  | 90.26-90.26 | 3.18 | 5.40 |
|  | *qRFW-20-2* | LG20 |  |  | 99.2-99.2 | 3.07 | 4.70 |
|  | *qRFW-26* | LG26 |  |  | 97.17-99.88 | 4.02 | 6.10 |
