# Supplementary material for: High-density genetic map construction and identification of loci controlling flower-type traits in Chrysanthemum (Chrysanthemum × morifolium Ramat.)
Source: Hortic Res. 2020 Jul 1;7:108. doi: 10.1038/s41438-020-0333-1 (PMC7326996; doi:10.1038/s41438-020-0333-1)
Supplement: Supplementary file 3 — Supplementary Table 3 [file 41438_2020_333_MOESM3_ESM.docx]

**Supplementary Table 3.** Annotation information of candidate gene based on sunflower genome

| Traits | Gene name | Annotation information |
| --- | --- | --- |
| CTMD | Ha4_00023899 | Cellular Component: spliceosomal complex  Biological Process: mRNA splicing, via spliceosome |
|  | Ha6_00049408 | Biological Process: PSII associated light-harvesting complex II catabolic process  Biological Process: chlorophyll catabolic process  Biological Process: oxidation-reduction process  Cellular Component: integral component of membrane |
|  | Ha17_00009700 | Molecular Function: porphobilinogen synthase activity  Biological Process: porphyrin-containing compound biosynthetic process  Molecular Function: metal ion binding |
| RNRF | Ha12_00034156 | Molecular Function: ATP binding  Biological Process: ATP catabolic process  Biological Process: ATP biosynthetic process  Biological Process: cation transport  Cellular Component: integral component of membrane  Molecular Function: cation-transporting ATPase activity  Molecular Function: metal ion binding |
|  | Ha8_00042732 | Unknown |
|  | Ha10_00002465 | Unknown |
|  | Ha10_00003279 | Molecular Function: binding  Cellular Component: plastid  Biological Process: cellular metabolic process |
|  | Ha11_00029340 | Molecular Function: protein kinase activity  Biological Process: phosphorylation |
