# Supplementary material for: High-density genetic map construction and identification of loci controlling flower-type traits in Chrysanthemum (Chrysanthemum × morifolium Ramat.)
Source: Hortic Res. 2020 Jul 1;7:108. doi: 10.1038/s41438-020-0333-1 (PMC7326996; doi:10.1038/s41438-020-0333-1)
Supplement: Supplementary file 4 — Supplementary Table 4 [file 41438_2020_333_MOESM4_ESM.docx]

**Supplementary Table 4.** Annotation information of candidate gene based on lettuce genome

| Traits | Scaffold name | Annotation information |
| --- | --- | --- |
| CTMD | FZNH01002288.1 | *Helianthus annuus* plasma membrane ATPase-like (LOC110911454), mRNA |
|  | FZNH01033998.1 | *Solanum lycopersicum* cultivar I-3 chromosome 12 |
|  | FZNH01002608.1 | *Helianthus annuus* probable chlorophyll(ide) b reductase NYC1, chloroplastic (LOC110865589), mRNA |
|  | FZNH01096507.1 | Putative ribonuclease H-like domain-containing protein [*Helianthus annuus*] |
|  | FZNH01026408.1 | *Helianthus annuus* peroxisome biogenesis protein 5 (LOC110942187), transcript variant X2, mRNA |
|  | FZNH01038845.1 | *Diplostephium hartwegii* voucher TEX:Vargas 456 mitochondrion, complete genome |
|  | FZNH01039533.1 | *Helianthus annuus* uncharacterized LOC110881398 (LOC110881398), mRNA |
|  | FZNH01054739.1 | *Ipomoea nil* 60S ribosomal protein L5-like (LOC109162615), mRNA |
| RNRF | FZNH01010880.1 | *Helianthus annuus* uncharacterized LOC110886753 (LOC110886753), transcript variant X8, ncRNA |
|  | FZNH01002288.1 | *Helianthus annuus* plasma membrane ATPase-like (LOC110911454), mRNA |
|  | FZNH01010252.1 | Phalaenopsis equestris switch 2 (LOC110023523), mRNA |
|  | FZNH01016120.1 | *Cucumis melo* genomic chromosome, chr_1  *Cucumis melo* genomic scaffold, anchoredscaffold00026 |
|  | FZNH01026408.1 | *Helianthus annuus* peroxisome biogenesis protein 5 (LOC110942187), transcript variant X2, mRNA  *Helianthus annuus* clone ZVG23_ZENA17-R ZVG23 locus putative peroxisomal targeting signal 1 receptor genomic sequence |
|  | FZNH01015848.1 | Unknown |
|  | FZNH01096507.1 | Unknown |
|  | FZNH01006755.1 | Unknown |
|  | FZNH01016711.1 | Unknown |
|  | FZNH01119316.1 | Unknown |
|  | FZNH01016554.1 | Unknown |
