# Supplementary figures and images for: High-density genetic map construction and identification of loci controlling flower-type traits in Chrysanthemum (Chrysanthemum × morifolium Ramat.)
Source: Hortic Res. 2020 Jul 1;7:108. doi: 10.1038/s41438-020-0333-1 (PMC7326996; doi:10.1038/s41438-020-0333-1)

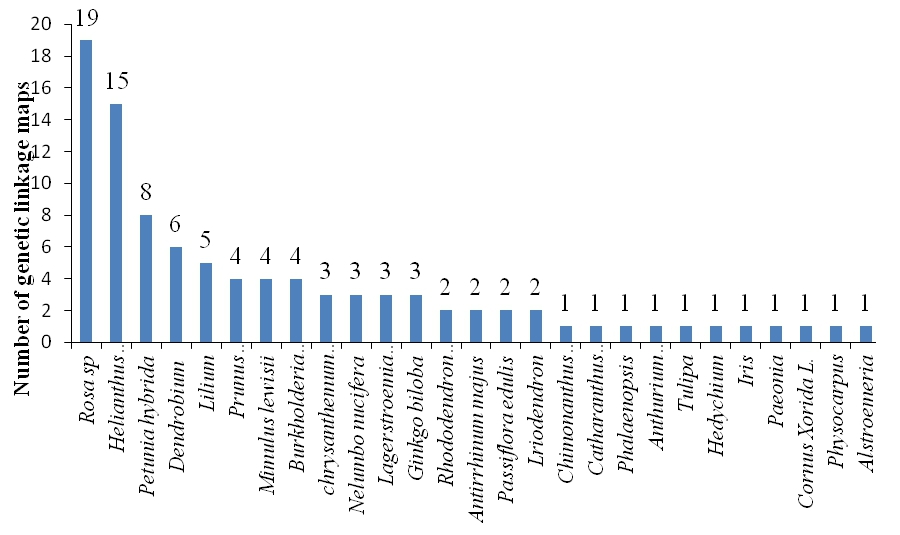

Supplement: Supplementary file 9 — Supplementary Figure 1 [file 41438_2020_333_MOESM9_ESM.jpg]

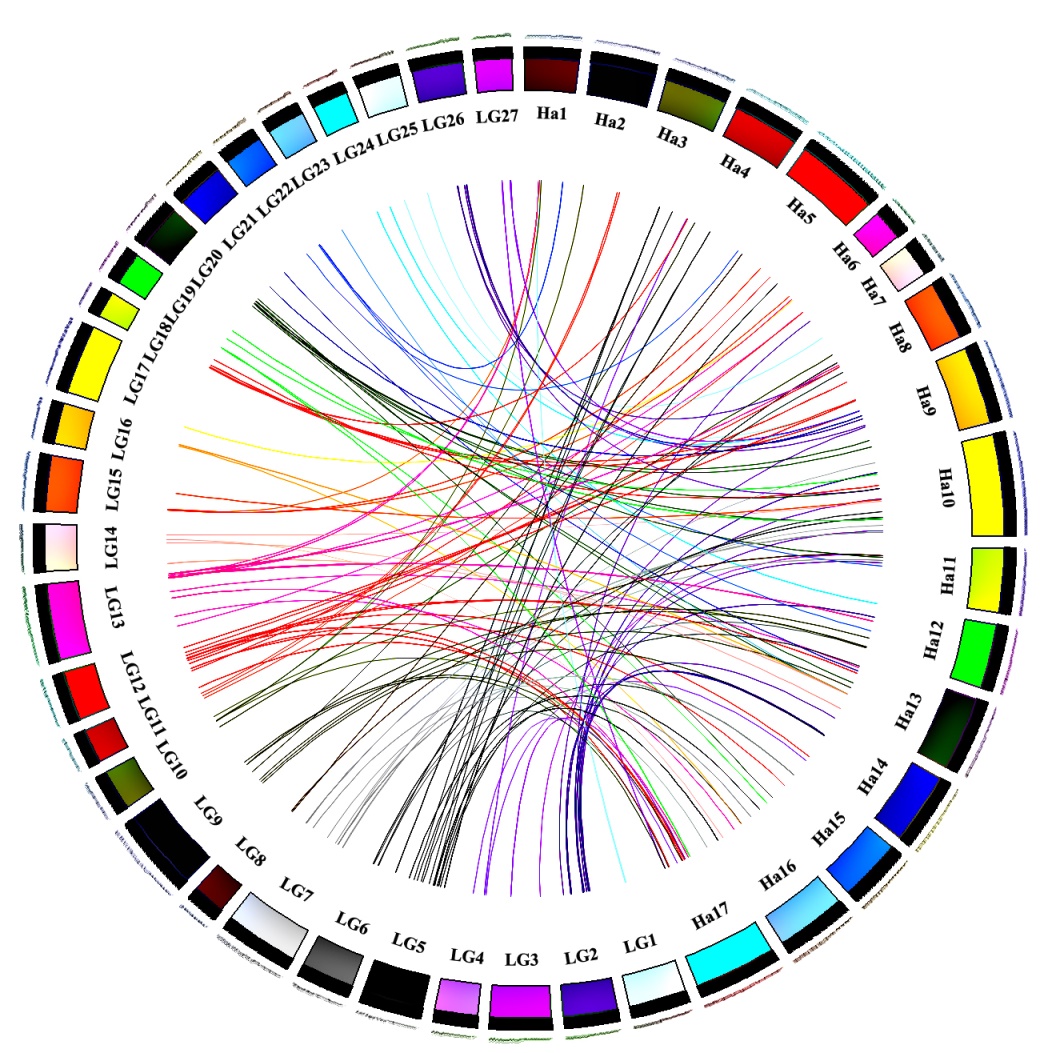

Supplement: Supplementary file 10 — Supplementary Figure 2 [file 41438_2020_333_MOESM10_ESM.jpg]

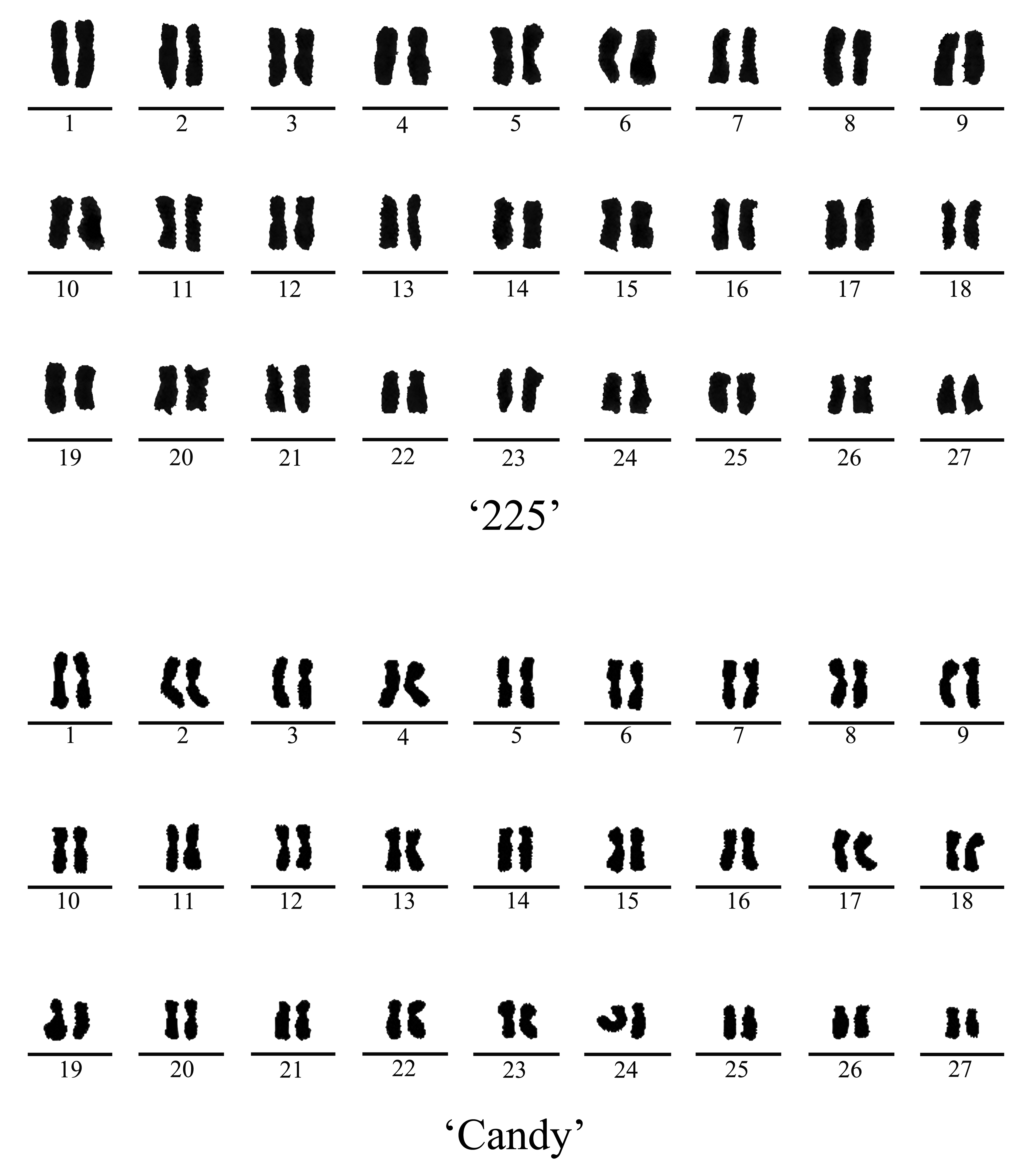

Supplement: Supplementary file 11 — Supplementary Figure 3 [file 41438_2020_333_MOESM11_ESM.jpg]

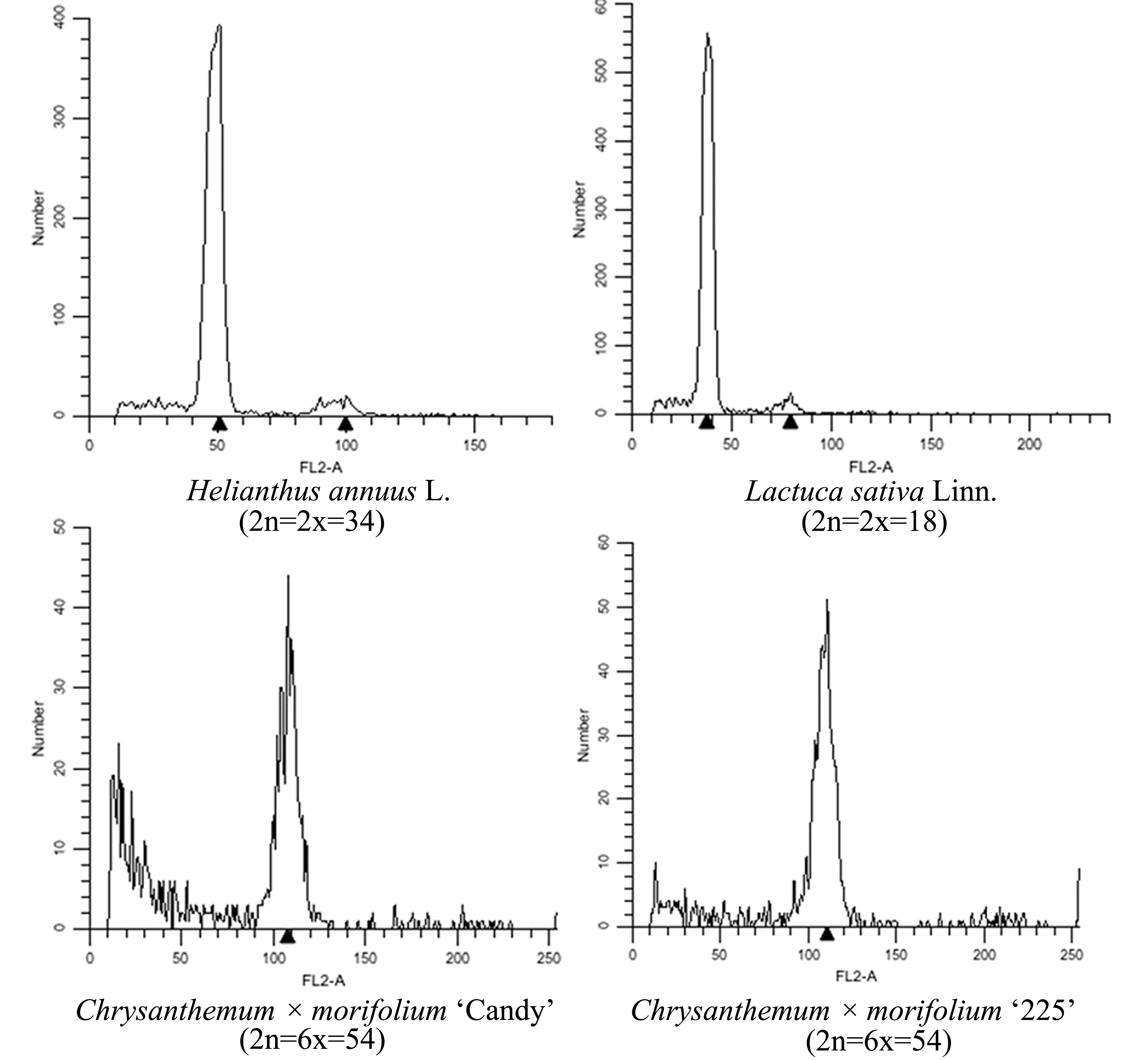

Supplement: Supplementary file 12 — Supplementary Figure 4 [file 41438_2020_333_MOESM12_ESM.jpg]
